# Feasibility of the Figure-of-8-Suture as Venous Closure in Interventional Electrophysiology: One Strategy for All?

**DOI:** 10.7150/ijms.42593

**Published:** 2020-04-06

**Authors:** Christoph J. Jensen, Miriam Schnur, Sebastian Lask, Philipp Attanasio, Michal Gotzmann, Kaffer Kara, Christoph Hanefeld, Andreas Mügge, Alexander Wutzler

**Affiliations:** 1 Cardiovascular Centre, St. Josef-Hospital, Ruhr-University Bochum, Germany; 2 Department of Internal Medicine and Cardiology, Charité University Medicine, Campus Benjamin Franklin, Berlin, Germany; 3 Department of Medicine III, St. Josef and St. Elisabeth Hospital, Bochum, Germany

## Abstract

**Background**: Venous vascular access with higher sheath size is common in interventional electrophysiology. In contrast to arterial vascular access, no dedicated closure devices exist for closure after venous access with higher sheath sizes. The Figure-of-8-Suture, an easy to apply suture, may be as a feasible approach for closure venous puncture. Our aim was to evaluate the feasibility of closure of femoral venous access.

**Methods**: From November 2016 to February 2018, patients undergoing electrophysiological procedures, closure of left atrial appendage or patent foramen ovale were included. Until May 2017, manual compression was performed to achieve haemostasis at venous access site (control group). From May 2017, patients were treated with a Figure-of-8-Suture (treatment group, Figure 1). Turnaround time and incidence of vascular complications were compared between the two groups.

**Results**: In total, 290 patients were included, 132 in the control group and 158 in the Figure-of-8-Suture group. Hemostasis after sheath removal was achieved in 100% of the cases in the control group by manual compression and in 98.7% of the cases with the Figure-of-8-Suture (p=0.2). Vascular complications were more common in the control group (6.8 vs. 1.3 %, p=0.01). Turnaround time was significantly lower in the Figure-of-8-Suture group (58.6 ± 14 vs. 77 ± 33.9 min, p=0.004).

In a sub-analysis in obese patients with body mass index (BMI) ≥30 kg/m^2^ (Figure-of-8 n=45, controls n=35), vascular complications were significantly more common in the control group (9.4 vs 0%, p=0.045).

**Conclusion**: The Figure-of-8-Suture is an easy-to-apply, effective approach for venous closure after electrophysiological procedures.

## Introduction

Venous vascular access with sheath size up to 14 French calibre is not uncommon in interventional electrophysiology and cardiology. Often, even double femoral access is necessary and periprocedural anticoagulation is administered. In contrast to arterial vascular access, where a variety of closure devices are available, no dedicated devices exist for closure after venous puncture. However, some studies report successful venous closure in sheath sizes up to 11 french with off-label use of arterial vascular closure devices^1^. Though, in clinical routine postprocedural vascular haemostasis is usually achieved by manual compression. Yet, vascular complications occur in a substantial number of patients undergoing electrophysiological procedures.^2^

Recently, alternative approaches to achieve venous haemostasis have been proposed for different types of procedures. Among those alternatives, the Figure-of-8-Suture, an easy to apply suture, seems to be a promising approach for closure after venous puncture for procedures in interventional cardiology^3^,^4^ or atrial fibrillation ablation.^5^ Our aim was to evaluate the feasibility and the impact on turnaround time, workflow improvement and patient satisfaction of the Figure-of-8-Suture as general approach for closure of femoral venous access in interventional electrophysiology.

## Methods

From November 2016 to February 2018, patients undergoing electrophysiological (EP) procedures, closure of left atrial appendage or closure of patent foramen ovale were included in the study. Until May 2017, manual compression was performed to achieve haemostasis at venous access site (control group) followed by an 8hour bed rest with groin compression bandage for 12 hours. After May 2017, patients were treated with a Figure-of-8-Suture (Figure-of-8-Suture group). Patients with additional or inadvertent arterial femoral vascular access or patients in whom vascular sheaths were not removed in the EP lab for other reasons (e. g. transfer to intensive care unit) were excluded from the study.

### Haemostasis in the control group

In the control group, after removal of all sheaths manual compression at the puncture site by a physician was used to achieve haemostasis. Manual compression was performed on the EP lab table before transferring the patient to the hospital bed. Activated Clotting Time (ACT) was measured at the end of the procedure before removal of the sheaths and Protamine was administered if ACT was above 300 s. After haemostasis was achieved, groin compression bandage was applied for at least 12 hours and 8 hours of bed rest were indicated. After removal of the compression bandage, clinical examination (inspection and auscultation) of the puncture site was performed by a physician.

### Haemostasis in the Figure-of-8-Suture group

**Figure 1:**
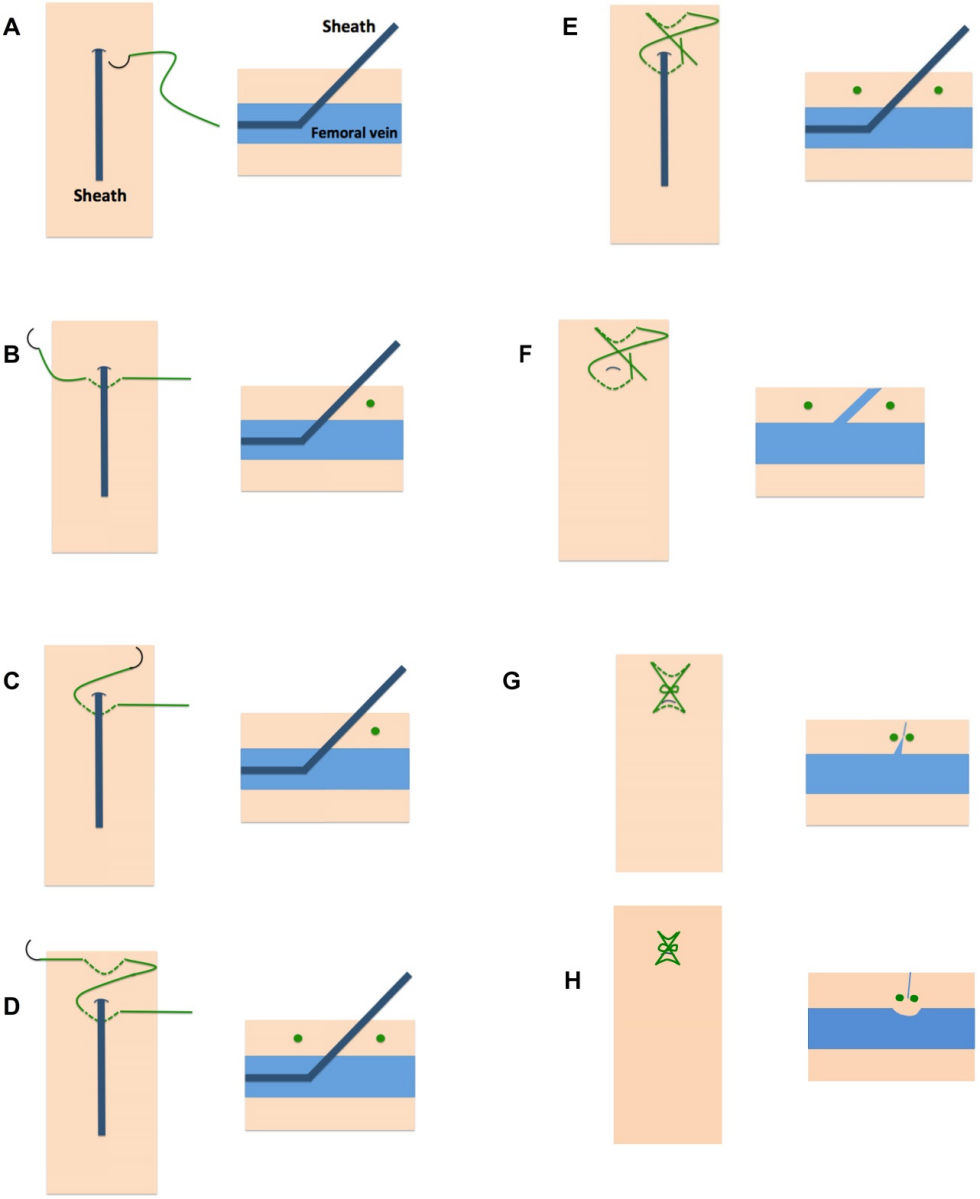
** Application of the Figure-of-8-Suture**. With the sheath in position (A) a 0 non-absorbable, braided polyester suture is passed caudal the skin puncture site under the sheath through subcutaneous tissue (B).After exiting the skin the needle is crossed over the sheath and enters the subcutaneous tissue cranial the skin puncture site in subcutaneous tissue above the sheath (C, D). During sheath removal a knot is tied with the two ends across the puncture (E, F, G). Final result shows the involution of the encompassed tissue by the suture which results in slight vasoconstriction of the vein and hence complete closure of the access site (H).

Before sheath removal a Figure-of-8-Suture was applied as described before.^5^ In brief, a 0 non-absorbable, braided polyester suture is passed caudal the skin puncture site under the sheath through subcutaneous tissue (Figure 1). After exiting the skin the needle is crossed over the sheath and enters the subcutaneous tissue cranial the skin puncture site in subcutaneous tissue above the sheath (Figure 1).^5^,^6^ During sheath removal a knot is tied with the two ends across the puncture. Due to a locking knot, traction was applied on the suture-of-eight to gather the encompassed tissue and create a mechanical tamponade effect on the venous puncture site. Additionally, a slight vasoconstriction of the vein occurs and hence facilitates complete closure of the access site (Figure 1).

In case of multiple puncture sites, every puncture site was treated with the Figure-of-8-Suture. In case of two adjacent small caliber punctures it was at the discretion of the interventional cardiologist to use one suture to close both puncture sites.

After application of the suture, groin compression bandage was applied for at least 12 hours and 8 hours of bed rest were indicated. Suture was removed after 4 hours. In contrast to the control group no patient received protamine before application of the figure-of-8-suture.

### Statistical analysis

Data are presented as absolute numbers and percentages for categorical variables or mean ± standard deviation (SD) for continuous variables. Study endpoint were incidence of vascular complications and turnover time. Vascular complications were defined as hematoma, pseudoaneurysms, fistulae and thrombosis. In cases of pseudoaneurysms with accompanying hematoma the complication was defined as pseudoaneurysm only to avoid double counts. Chi square test was used to compare discrete variables, Mann Whitney u test was used to compare continuous variables. All analyses were performed using SPSS software version 24.0 (SPSS Inc., Chicago, IL, USA). A p value of < 0.05 was considered statistically significant.

## Results

During the study period, 290 patients were included, 132 in the control group and 158 in the Figure-of-8-Suture group (Table 1). Age, gender, comorbidities and use of anticoagulant and antiplatelet therapy were not significantly different between the groups. Sheath size at suture site was 14.9 ± 2.9 Fr in the control group and 14.8 ± 2.8 in the treatment group (p=0.2). Hemostasis after sheath removal was achieved in 100% of the cases in the control group by manual compression and in 98.7% of the cases with the Figure-of-8-Suture (p=0.2, table 2). In two patients, in whom hemostasis could not be established with the Figure-of-8-Suture (rupture of the suture in one case and bleeding despite the application of a suture in one case), hemostasis was achieved with manual compression. Turnaround time (time from removal of the sheaths to puncture of the next patient) was significantly lower in the Figure-of-8-Suture group (58.6 ± 14 vs. 77 ± 33.9 min, p=0.004). Incidence of vascular complications was significantly lower in the Figure-of-8-suture group compared to manual compression (6.8 vs. 1.3 %, p=0.01).

In a sub-analysis, all obese patients were identified. Obesity was defined as body mass index (BMI) ≥30 kg/m^2^. Forty-five obese patients in the Figure-of-8-Suture group were compared to 35 patients of the control group (Table 3). Clinical characteristics were not significantly different between the sub-groups of obese patients. Yet, vascular complications were significantly more frequent in the control group (9.4 vs 0%, p=0.045; Table 3).

## Discussion

We here present a study on the use of a Figure-of-8-Suture as closure after venous vascular access as a general approach in interventional electrophysiology. Our data show that the Figure-of-8-Suture is feasible and associated with a low complication rate. Turnaround time was significantly reduced in cases with a Figure-of-8-Suture.

Our study evaluated the Figure-of-8-Suture for different types of electrophysiological procedures. Our results confirm data from atrial fibrillation ablation procedures^5^ and procedures in interventional cardiology.^3^,^4^ Groin complications are amongst the most frequent complications in cardiology.^2^ Complication rate was low in Figure-of-8-Suture group. Yet, two patients developed a groin hematoma despite a Figure-of-8-Suture. In the first patient, the suture was ruptured and acute hemostasis was achieved with manual compression. The second patient developed a groin hematoma after removal of the suture. Bleeding and hematoma after Figure-of-8-Suture have been reported before.^3^ However, incidence was significantly lower compared to manual compression in previous studies and were rare events as in our study.^3^

The procedures that were performed differed slightly between our study groups (table 2). Yet, neither sheath diameter nor use of anticoagulants or antiplatelet therapy was significantly different between the two groups.

It has been pointed out by several authors, that optimal workflow is one of the biggest challenges in healthcare organizations.^7^,^8^ Turnaround time is crucial for the optimization of and patient satisfaction.^7^,^8^,^9^,^10^ Improvement of workflow in the catheterization laboratory is therefore desirable. The results of our study show, that the Figure-of-8-Suture may serve as a time saving, feasible method of venous closure in interventional electrophysiology.

An interesting aspect is, that our results were reproducible in a subgroup of obese patients. Noteworthy, in our sub-analysis the mean BMI was 35 in the Figure-of-8-Suture group and the control group. A BMI of 35 defines Grade 2 obesity.^11^ Despite the relatively high BMI in our sub-groups, the Figure-of-8-Suture was applied successfully in all cases. Previous studies revealed increased rate of minor^12^ and vascular complications^13^ in obese patients during electrophysiological procedures with venous access. In our study, the Figure-of-8-Suture was feasible and associated with a lower rate of vascular complication compared to manual compression. Therefore, the Figure-of-8-Suture may facilitate venous closure also in obese patients in the future.

### Limitations

There are several limitations that should be acknowledged. First, this was a single centre study. Secondly, patients were recruited in a non-randomized fashion. Furthermore, the procedures differed slightly between the groups. For the ease of study flow, no recording of the mean procedure times was performed. Differences in mean procedure times could have influenced the bleeding risk at puncture sites. However, neither sheath size nor percentage of patients under chronic anticoagulation were significantly different between the groups.

Our study should be considered as a pilot study and the results should be confirmed in a lager study cohort.

## Conclusion

The Figure-of-8-Suture is an easy-to-apply, effective approach for venous closure after electrophysiological procedures.

## Figures and Tables

**Table 1:** Baseline characteristics.

	Control (n=132)	Figure of 8 suture (n=158)	*P* value
**Characteristics**			
Age (years)	66 ± 10.9	66.4 ± 13.2	0.3
Male (%)	77 (58.3)	106 (67.1)	0.12
BMI (kg/m^2^)	29 ± 5.3	28.7 ± 5.6	0.7
LVEF (%)	53.5 ± 9.1	51.6 ± 10.1	0.3
Hypertension (%)	88 (66.7)	114 (72.2)	0.3
CAD (%)	29 (22)	50 (31.6)	0.07
Diabetes mellitus (%)	13 (9.8)	28 (17.7)	0.06
PAD (%)	12 (9.1)	9 (5.7)	0.3
Oral anticoagulation (%)	99 (75)	115 (72.8)	0.7
Aspirin (%)	24 (18.2)	39 (24.7)	0.2
Clopidogrel (%)	10 (7.6)	17 (10.8)	0.4
**Procedures**			
Pulmonary vein isolation (%)	70 (53)	67 (42.4)	0.07
Ablation of atrial flutter (%)	36 (27.3)	35 (22.2)	0.33
Ablation of SVT (%)	3 (2.3)	19 (12)	0.002*
VT ablation (%)	22 (16.7)	22 (13.9)	0.52
LAA/PFO closure (%)	1 (0.8)	10 (6.3)	0.013*
Electrophysiological study (%)	0	5 (3.2)	0.04

BMI = body mass index, CAD = coronary artery disease, LVEF = left ventricular ejection fraction, PAD = peripheral arterial disease. *statistically significant

**Table 2:** Procedures, turnover time and incidence of vascular complication compared between the groups.

	Control (n=132)	Figure of 8 suture (n=158)	*P* value
Sheath size at suture site (French)	14.9 ± 2.9	14.8 ± 2.8	0.2
Haemostasis achieved (%)	132 (100)	156 (98.7)	0.2
Turnaround time^a^ (min)	77 ± 33.9	58.6 ± 14	0.004*
Vascular complication (%)	9 (6.8)	2 (1.3)	0.01*
Haematoma (%)	3	2	
Pseudoaneurysm (%)	4	-	
Fistula (%)	2	-	
Thrombosis (%)	-	-	

^a^time from sheaths removal to puncture of the next patient. *statistically significant.

**Table 3:** Characteristics and complications of obese patients (body mass index ≥30 kg/m^2^).

	Control (n=35)	Figure of 8 suture (n=45)	*P* value
**Age (years)**	63.3 ± 11.2	65.1 ± 12.1	0.4
**Male (%)**	23 (65.7)	30 (66.7)	0.9
**BMI (kg/m^2^)**	35.6 ± 3.1	35.4 ± 4.7	0.8
**LVEF (%)**	54.2 ± 9.9	52 ± 10	0.2
**Hypertension (%)**	31 (88.6)	36 (80)	0.3
**CAD (%)**	8 (22.9)	15 (33.3)	0.3
**Diabetes mellitus (%)**	6 (17.1)	11 (24.4)	0.4
**PAD (%)**	3 (9.4)	2 (4.4)	0.4
**Oral anticoagulation (%)**	32 (91.4)	38 (84.4)	0 3
**Aspirin (%)**	4 (11.4)	5 (11.1)	1
**Clopidogrel (%)**	1 (2.9)	2 (4.4)	0.7
**Sheath size at suture site (French)**	16.1 ± 1.4	15.9 ± 0.9	0.05
**Haemostasis achieved (%)**	35 (100)	45 (100)	-
**Turnaround time^a^ (min)**	47.3 ± 25.5	55.2 ± 9.3	0.9
**Vascular complication (%)**	3 (9.4)	0	0.045*
Haematoma (%)	1	-	
Pseudoaneurysm (%)	2	-	
Fistula (%)	-	-	
Thrombosis (%)	-	-	

BMI = body mass index, CAD = coronary artery disease, LVEF = left ventricular ejection fraction, PAD = peripheral arterial disease. ^a^time from sheaths removal to puncture of the next patient. *statistically significant
